# Evaluating Uptake of Evidence-Based Interventions in 355 Clinics Partnering With the Colorectal Cancer Control Program, 2015–2018

**DOI:** 10.5888/pcd19.210258

**Published:** 2022-05-19

**Authors:** Annette E. Maxwell, Amy DeGroff, Sarah D. Hohl, Krishna P. Sharma, Juzhong Sun, Cam Escoffery, Peggy A. Hannon

**Affiliations:** 1 University of California Los Angeles, Los Angeles, California; 2 Centers for Disease Control and Prevention, Atlanta, Georgia; 3 University of Washington, Seattle, Washington; 4 Emory University, Atlanta, Georgia

## Abstract

**Purpose and Objectives:**

Colorectal cancer screening rates remain suboptimal in the US. The Colorectal Cancer Control Program (CRCCP) of the Centers for Disease Control and Prevention (CDC) seeks to increase screening in health system clinics through implementation of evidence-based interventions (EBIs) and supporting activities (SAs). This program provided an opportunity to assess the uptake of EBIs and SAs in 355 clinics that participated from 2015 to 2018.

**Intervention Approach:**

The 30 funded awardees of CRCCP partnered with clinics to implement at least 2 of 4 EBIs that CDC prioritized (patient reminders, provider reminders, reducing structural barriers, provider assessment and feedback) and 4 optional strategies that CDC identified as SAs (small media, professional development and provider education, patient navigation, and community health workers).

**Evaluation Methods:**

Clinics completed 3 annual surveys to report uptake, implementation, and integration and perceived sustainability of the priority EBIs and SAs.

**Results:**

In our sample of 355 clinics, uptake of 4 EBIs and 2 SAs significantly increased over time. By year 3, 82% of clinics implemented patient reminder systems, 88% implemented provider reminder systems, 82% implemented provider assessment and feedback, 76% implemented activities to reduce structural barriers, 51% implemented provider education, and 84% used small media. Most clinics that implemented these strategies (>90%) considered them fully integrated into the health system or clinic operations and sustainable by year 3. Fewer clinics used patient navigation (30%) and community health workers (19%), with no increase over the years of the study.

**Implications for Public Health:**

Clinics participating in the CRCCP reported high uptake and perceived sustainability of EBIs that can be integrated into electronic medical record systems but limited uptake of patient navigation and community health workers, which are uniquely suited to reduce cancer disparities. Future research should determine how to promote uptake and assess cost-effectiveness of CRCCP interventions.

**Summary**

**What is already known about the topic?**

Evidence-based interventions, such as patient and provider reminders, provider assessment and feedback, small media, and patient navigation are effective in increasing colorectal cancer screening.

**What is added by this report?**

Our evaluation of primary care clinics that participated in the Centers for Disease Control and Prevention’s Colorectal Cancer Control Program showed a high uptake of interventions that can be integrated into electronic medical records but limited uptake of patient navigation and community health workers.

**What are the implications for public health practice?**

Future research should further explore the use of patient navigation and community health workers in colorectal cancer prevention because these strategies are uniquely suited to reducing cancer disparities.

## Introduction

Screening reduces deaths related to colorectal cancer (CRC), the second-leading cause of cancer death in the US (1). However, despite recommendation by the US Preventive Services Task Force, CRC screening rates remain suboptimal (66% in 2018) (2); rates among uninsured and low-income populations are even lower. For example, in 2018, only about 30% of people who were uninsured and fewer than 50% of individuals who received care at Federally Qualified Health Centers, government-supported safety net clinics, were up to date with CRC screening (2,3). 

The Community Preventive Services Task Force oversees rigorous, systematic reviews of the scientific literature to identify prevention strategies with evidence of effectiveness. On the basis of these reviews, the Task Force recommends the following evidence-based interventions (EBIs) to increase CRC screening: patient reminders, provider reminders, reducing structural barriers, provider assessment and feedback, small media, one-on-one education, and community health workers, including patient navigators (4) (Table 1). Few studies have evaluated the uptake and sustainability of EBIs in a large sample of health care clinics (5,6). Such data are needed to understand how these interventions affect population health, as well as how best to increase the scale of effective interventions. Scalability is defined as the ability of an efficacious health intervention to be expanded under real-world conditions to reach a large proportion of the eligible population (7).

**Table 1:** Definitions of Evidence-Based Interventions and Supporting Activities in the CDC Colorectal Cancer Control Program Clinic Survey, 2015–2018

Evidence-based interventions	Definitions provided to participants
Patient reminder system	System to remind patients when they are due for screening that is in written form (letter, postcard, email) or by telephone voice messages (including automated messages).
Provider reminder system	System to inform providers that a patient is due (or overdue) for screening. Reminders can be provided in different ways, such as in patient charts or by email.
Provider assessment and feedback	System to both evaluate provider performance in delivering or offering screening to clients (assessment) and present providers with information about their performance in providing screening services (feedback).
Reducing structural barriers	Clinic has assessed structural barriers to colorectal cancer screening and has addressed barriers through 1 or more interventions. Structural barriers are noneconomic burdens or obstacles that make it difficult for people to access cancer screening. Reducing structural barriers does not include patient navigation or community health workers.
**Supporting activities**	
Small media	Materials used to inform and motivate people to be screened for cancer, including videos and printed materials, letters, brochures, and newsletters.
Professional development and provider education	Activities may include distribution of provider education materials, including screening guidelines and recommendations, or continuing medical education opportunities.
Community health workers	Lay health educators with a deep understanding of the community who are often from the community being served. Community health workers work in community settings, in collaboration with a health promotion program, clinic, or hospital, to educate people about cancer screening, promote cancer screening, and provide peer support to people referred to cancer screening.
Patient navigation	Patient navigators typically assist clients in overcoming individual barriers to cancer screening. Patient navigation includes assessment of client barriers, client education and support, resolution of client barriers, client tracking, and follow-up. Patient navigation should involve multiple contacts with a client.

Abbreviation: CDC, Centers for Disease Control and Prevention.

In 2015, the Centers for Disease Control and Prevention (CDC) funded the Colorectal Cancer Control Program (CRCCP) with the goal of increasing CRC screening. Thirty awardees were required to partner with primary care clinics that serve high-need populations to implement EBIs to increase CRC screening. On the basis of recommendations from the Task Force, CDC named 4 EBIs as priority for implementation (patient reminders, provider reminders, reducing structural barriers, provider assessment and feedback). These 4 priority EBIs can be implemented at the health system level to change screening rates. CDC deemed the 4 other EBIs that focus on the individual level (small media, one-on-one education, community health workers, patient navigators) as optional supporting activities (SAs). Awardees could implement both EBIs and SAs.

## Purpose and Objectives

The primary purpose of this analysis was to determine the uptake and sustainability of EBIs and SAs in clinics participating in the CRCCP program over 3 years, from 2015 to 2018. We define uptake as the initial decision to employ an EBI or SA in a clinic setting (also called adoption), while sustainability indicates integration of an EBI or SA into a clinic’s ongoing operation (8). With regard to SAs, we were especially interested in the uptake of patient navigation by these clinics because most clinics in the CDC program are Federally Qualified Health Centers that provide care to underserved and under-resourced populations that experience health disparities, and patient navigation is a strategy intended to reduce disparities by helping patients overcome barriers to health care (9). Patient navigation is well accepted in these populations (10–13) and can be integrated into existing roles in clinical settings (14–16). The Task Force recently added patient navigation, conducted by patient navigators or community health workers, to their list of recommended interventions to promote CRC screening because it increases CRC screening rates (11,17). CDC defines patient navigation for CRC screening as individualized assistance offered to patients to help address barriers and facilitate timely access to quality screening and follow-up, as well as initiation of treatment services for people diagnosed with cancer. Patient navigation includes assessment of patient barriers, patient education, resolution of barriers, and patient tracking and follow-up. Patient navigation can be provided by health care providers (eg, nurses) or lay workers (eg, community health workers) (18).

## Intervention Approach

The CRCCP uses a 5-year funding cycle, and our analysis focused on the 2015 through 2020 cycle. The 30 funded awardees partnered with clinics and provided technical assistance and resources to implement Task Force–recommended EBIs. For this screening program, awardees were required to implement at least 2 of the CDC-prioritized EBIs, as well as SAs; however, awardees were not allowed to use SAs as stand-alone activities. In addition, small media, in particular, had to be paired with 1 of the 4 EBIs (eg, a mailed patient reminder could include a small media material). The screening program is based on several tenets, including integrating public health and primary care, focusing on populations with a high prevalence of disease, implementing sustainable health system changes, and using evidence-based approaches to maximize limited public health dollars (18,19). CRCCP provided an opportunity to study the uptake and sustainability of different EBIs and SAs in a large number of health system clinics that provide care to medically underserved patients and to consider their scalability. Previous studies of this program observed that the implementation of its strategies was associated with increased clinic-level screening rates (18,20).

## Evaluation Methods

CDC’s Framework for Program Evaluation was applied to design the clinic survey on which this analysis is based (21). Other components of the CRCCP evaluation include an annual survey of awardees (22), cost effectiveness studies (23), case studies, and studies to explore specific components of CRCCP (18,24).

The clinic survey was based on prior surveys (5,25) and was completed by 1 representative per clinic, similar to other studies (25,26). Data collected in the surveys included clinic characteristics such as clinic type and size, EBIs and SAs in place at baseline and annually, use of CDC resources (eg, staff time, funds, materials) toward implementing EBIs and SAs, sustainability of EBIs and SAs, and baseline and annual CRC screening rates (21). Uptake was defined as EBIs and SAs that are in place and operational (in use) in a clinic at the end of the reporting period. Respondents were asked about sustainability using the question, “If in place, do you consider the EBI or SA as fully integrated into health system or clinic operations and sustainable?” “High quality implementation has been achieved and a supporting infrastructure is in place along with any financial support needed to maintain the EBI/SA. The EBI/SA has become an institutionalized component of the health system and/or clinic operation” was provided as an explanation. Respondents were not asked to consider the length of time that the strategy had been implemented in their responses. Definitions for EBIs and SAs that were given to survey respondents are provided (Table 1). Awardees compiled and reported data to CDC from annual clinic surveys for each participating clinic for each of the first 3 years, from 2015 to 2018.

### Statistical analysis

The study sample was limited to clinics that enrolled in the first year of CDC’s screening program (2015–2016) and remained in the program for 3 years (N = 355 clinics, 85% of 417 clinics enrolled). We conducted a descriptive analysis to 1) identify the proportion of clinics implementing the 4 priority EBIs and 4 SAs for each year of the study period and 2) assess whether the EBIs and SAs were perceived as integrated and sustainable by the end of the study period. For each EBI and SA, trends in use between baseline and year 3 were analyzed by using the Cochran–Armitage test for trend. Analyses were conducted using SAS software, version 9.4 (SAS Institute Inc).

## Results

### Clinic characteristics

Most clinics were Federally Qualified Health Centers (73%), and clinic size varied. Some clinics had fewer than 500 patients aged 50 to 75 years (24%), and others had more than 1,500 patients (38%). The number of providers ranged from fewer than 5 providers per clinic (42%) to more than 20 providers (12%). Patient populations ranged from less than 5% uninsured, aged 50 to 75 (29% of clinics) to more than 20% uninsured patients (36% of clinics). Thirty-four percent of all clinics had access to free fecal testing kits. Most clinics used stool-based tests as their primary CRC screening test (56%); 29% referred patients for colonoscopy, and in 13% of clinics, the primary screening test varied by provider (Table 2).

**Table 2:** Characteristics of Clinics Partnering With the CDC Colorectal Cancer Control Program Evaluation (N = 355), 2015–2018

**Characteristics**	**N (%)**
Federally Qualified Health Center or community health center	258 (72.7)
Health system–owned or hospital-owned	49 (13.8)
Health department, tribal health center, or other	32 (9.0)
Private or physician owned	16 (4.5)
Number of clinic patients aged 50–75 y	
<500	85 (23.9)
500–1,500	137 (38.6)
>1,500	133 (37.5)
Number of primary care providers	
<5	150 (42.3)
5–20	159 (44.8)
>20	44 (12.4)
Missing	2 (0.5)
Percentage of uninsured patients aged 50–75 y	
<5	104 (29.3)
5–20	94 (26.5)
>20	129 (36.3)
Missing	28 (7.9)
Access to free fecal testing kits	
Yes	121 (34.1)
No	209 (58.9)
Unknown	25 (7.0)
Type of primary colorectal cancer screening tests	
Stool-based tests	197 (55.5)
Colonoscopy referral	103 (29.0)
Varies by provider	47 (13.2)
Unknown	8 (2.3)

### Uptake of strategies to promote CRC screening

Uptake of strategies to promote CRC screening among clinics varied widely at baseline and throughout the study. At baseline, 50% of clinics used patient reminder systems, 72% implemented provider reminder systems, 50% used provider assessment and feedback, and 43% implemented activities to reduce structural barriers. Significant increases were observed in the uptake of all 4 EBIs in the first 3 years of the program (*P* < .001 for all 4 EBIs). In year 3, 82% of clinics implemented patient reminder systems, 88% implemented provider reminder systems, and 82% implemented provider assessment and feedback. At baseline, SA use was generally low; 17% of clinics used community health workers, 32% offered patient navigation, 36% used small media, and 43% delivered provider education. Among SAs, professional development and provider education increased significantly, from 43% to 51% (*P* = .001), and use of small media increased significantly, from 36% to 84% (*P* < .001) of clinics in year 3 (Figure and Table 3).

**Figure:**
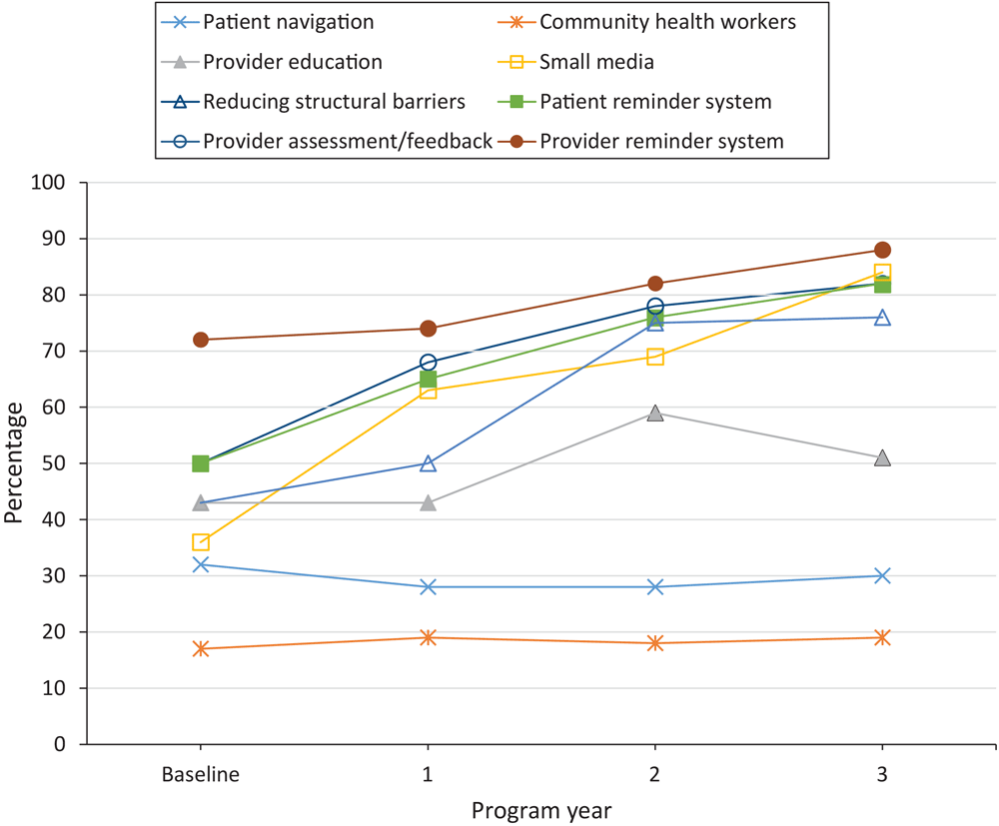
Percentage of clinics that partnered with the CDC Colorectal Cancer Control Program using evidence-based interventions to promote colorectal cancer screening, analyzed using the Cochran–Armitage trend test, 2015–2018 (N = 355).

**Table:** 

Assessment years	Patient navigation	Community health workers	Provider education	Small media	Reducing structural barriers	Patient reminder system	Provider assessment and feedback	Provider reminder system
Baseline	32	17	43	36	43	50	50	72
Year 1	28	19	43	63	50	65	68	74
Year 2	28	18	59	69	75	76	78	82
Year 3	30	19	51	84	76	82	82	88

**Table 3:** Use of Priority Evidence-Based Interventions and Supporting Activities in Clinics Partnering With the CDC Colorectal Cancer Control Program (N = 355), 2015–2018

Evidence-based intervention type	Program year	Clinics that changed evidence-based interventions use from prior year^a^, N (%)	Evidence-based interventions in place^b^	
			N (%)	*P* value for trend^c^
Patient reminder system	Baseline	NR	177 (50)	<.001
	Year 1	128 (36)	231 (65)	
	Year 2	86 (24)	271 (76)	
	Year 3	45 (13)	290 (82)	
Provider reminder system	Baseline	NR	254 (72)	<.001
	Year 1	96 (27)	262 (74)	
	Year 2	54 (15)	290 (82)	
	Year 3	45 (13)	311 (88)	
Provider assessment and feedback	Baseline	NR	178 (50)	<.001
	Year 1	129 (36)	243 (68)	
	Year 2	61 (17)	276 (78)	
	Year 3	66 (19)	290 (82)	
Reducing structural barrier activities	Baseline	NR	153 (43)	<.001
	Year 1	121 (34)	176 (50)	
	Year 2	129 (36)	265 (75)	
	Year 3	56 (16)	269 (76)	
Patient navigation	Baseline	NR	114 (32)	.53
	Year 1	91 (26)	101 (28)	
	Year 2	88 (25)	101 (28)	
	Year 3	35 (10)	106 (30)	
Community health workers	Baseline	NR	60 (17)	.52
	Year 1	45 (13)	69 (19)	
	Year 2	14 (4)	63 (18)	
	Year 3	12 (3)	69 (19)	
Professional development and provider education	Baseline	NR	152 (43)	<.001
	Year 1	153 (43)	151 (43)	
	Year 2	90 (25)	211 (59)	
	Year 3	83 (23)	182 (51)	
Small media	Baseline	NR	127 (36)	<.001
	Year 1	160 (45)	225 (63)	
	Year 2	65 (18)	246 (69)	
	Year 3	73 (21)	297 (84)	

Abbreviations: CDC, Centers for Disease Control and Prevention; NR, not reported.

a Clinics that implemented or resumed evidence-based interventions and support activities that were not in place in the prior year or that paused or discontinued those interventions and activities that were in place in the prior year.

b Indicates evidence-based interventions and support activities are in place and operational (in use) in clinics at the end-of-program year, regardless of the quality, reach, or current level of functionality.

c Two-sided *P* value, Cochran–Armitage trend test.

A substantial number of clinics implemented or resumed new strategies and discontinued or paused strategies during the study period (Table 3). Overall, the proportion of clinics that changed their EBI use from the prior year ranged from 27% to 36% in the first year. These fluctuations tended to decrease in subsequent years, from 13% to 19% of clinics in year 3. SA implementation fluctuated similarly. Only 28% of clinics had patient navigation in place in the second and third year (after baseline) of the program, but almost the same proportion, 25% to 26% of clinics either newly implemented or discontinued patient navigation in the same program years. In the third year, the proportion of clinics that changed their patient navigation status (either new or discontinued) decreased to 10%.

 In the first year of the program, clinics that implemented 2 EBIs (n = 64) also implemented on average 1.4 SAs; those that implemented 3 EBIs (n = 102) implemented an average 1.7 SAs, and those that implemented 4 EBIs (n = 110) implemented an average 2.1 SAs. Concurrent implementation of EBIs and SAs was very similar in all program years. Not all clinics, however, implemented 2 priority EBIs in the first year. The percentage of clinics that implemented fewer than 2 EBIs ranged from 22% in the first year to 11% in the second year and to 4% in the third year. 

### Integration of strategies to promote CRC screening and sustainability

Among clinics that had EBIs and SAs in place by the end of each year, most considered those EBIs and SAs fully integrated into health systems or clinic operations and sustainable with or without CRCCP resources, especially in years 2 and 3 (Table 4). Sustainability and integration into clinic operations during the 3-year period increased most for activities that largely focused on providers, such as provider reminder systems, an increase of 14 percentage points from 79% in year 1 to 93% in year 3. Similarly, full integration of provider assessment and feedback increased 27 percentage points, from 69% to 96% of clinics; full integration of professional development or provider education increased by 16 percentage points, from 76% to 92% of clinics, followed by full integration of small media for an increase of 11 percentage points from 81% to 92% of clinics. Sustainability and integration into clinic operations did not substantially change with patient navigation (5 percentage point increase from 87% to 92% of clinics) and for community health workers (a 3 percentage point decrease, from 99% to 96%) (Table 4).

**Table 4:** Integration and Perceived Sustainability of Priority Evidence-Based Interventions and Supporting Activities in Clinics Partnering With the CDC Colorectal Cancer Control Program (CRCCP) (N = 355), 2015–2018

Intervention	Clinics that have specific EBI/SA in place^a ^by end of program year, N (%)	Fully integrated EBI/SA in place^b^		
		Yes, with or without CRCCP resources %	No, %	Unknown or missing, %
**Priority EBIs**				
Patient reminder systems				
Year 1	231 (65)	84	13	3
Year 2	271 (76)	95	3	2
Year 3	290 (82)	93	3	4
Provider reminder systems				
Year 1	262 (74)	79	13	8
Year 2	290 (82)	94	4	2
Year 3	311 (88)	93	3	4
Provider assessment and feedback				
Year 1	243 (68)	69	17	14
Year 2	276 (78)	93	3	4
Year 3	290 (82)	96	1	3
Activities to reduce structural barriers				
Year 1	176 (50)	91	2	7
Year 2	265 (75)	97	0	3
Year 3	269 (76)	98	1	1
**Supporting activities**				
Patient navigation				
Year 1	101 (28)	87	7	6
Year 2	101 (28)	93	0	7
Year 3	106 (30)	92	3	5
Community health workers				
Year 1	69 (19)	99	0	1
Year 2	63 (18)	98	0	2
Year 3	69 (19)	96	0	4
Professional development and provider education				
Year 1	151 (43)	76	15	9
Year 2	211 (59)	88	0	12
Year 3	182 (51)	92	0	8
Small media				
Year 1	225 (63)	81	12	7
Year 2	246 (69)	96	0	4
Year 3	297 (84)	92	2	6

Abbreviations: CRCCP, Colorectal Cancer Control Program; EBI, evidence-based interventions; SA, supporting activities.

a Indicates whether EBI/SA are in place by end of program year, regardless of quality, reach, or level of functionality.

b Indicates whether EBI/SA are fully integrated (institutionalized) by end of program year into the health system or clinic operations with supporting infrastructure and financial support to maintain the EBI/SA.

## Implications for Public Health

To our knowledge, this is one of only a few studies examining the uptake of evidence-based interventions to promote CRC screening in a large sample of clinics in 30 states. A 2012 study of 44 Federally Qualified Health Centers in 4 Midwestern states found that 41% of clinics had no CRC screening tracking system, although 79% reported using electronic health records (25). A 2016 cross-sectional survey of 56 Federally Qualified Health Centers in 7 states found that 73% of them implemented patient reminder systems, 77% implemented provider reminder systems, and 82% implemented provider assessment and feedback. The same study found that fewer clinics used patient navigators (50%) and small media (62%) (26). Our study builds on previous research in 3 ways: 1) by corroborating results regarding the implementation of these strategies, 2) by adding information on the uptake of 8 different EBIs and SAs, and 3) by assessing these strategies, their changes in implementation, and their sustainability and integration over a 3-year period in a large sample of clinics.

Overall, we observed significant uptake of 4 priority EBIs and 2 SAs, suggesting that the CRCCP contributed to increasing implementation of these strategies in the participating clinics. Our data suggest that for all strategies experimentation took place in early years of the program until clinics settled on strategies that worked for their particular contexts. In addition, many clinics required more than a year to implement at least 2 priority EBIs. Clinics that implemented fewer EBIs also tended to implement fewer SAs, and vice versa.

The strong and consistent uptake of priority EBIs by CRCCP clinics may exist, in part, because CDC requires that clinics implement 2 of the 4 priority EBIs, awardees provide technical assistance and implementation support to clinics, and for some clinics, financial support is provided by awardees. Another explanation is that some EBIs and SAs can be integrated into clinical practice through clinics’ electronic health records systems. For example, by using data from electronic health records, patient reminder letters can be generated and personalized with each patient’s name and address, preferred language, the name of the patient’s primary care provider, and their history of CRC screening (eg, type and time of most recent test). Although it takes resources to program electronic health records and to set up these strategies initially, clinic health information technology and automated calling and texting systems can support implementation (27,28). Whether clinics can maintain these interventions solely with their own resources after CRCCP technical and financial support has ended remains to be seen.

Implementation of patient navigation and use of community health workers, on the other hand, was much lower than the priority EBIs and remained low over time. CDC’s focus on the 4 priority EBIs and on sustainability could be the reason for low implementation of these activities. Patient navigation is resource intensive, requiring ongoing funding and dedicated staff. In one study, trained nurse navigators spent an average of 124 minutes per patient to deliver a 6-step protocol by telephone to navigate patients for colonoscopy (29). In addition, the costs of patient navigation can be substantial. An economic analysis of detailed activity-based cost information that was systematically collected in a subset of CRCCP clinics showed costs per person screened ranging from $24 to $40 in 14 clinics that implemented multicomponent interventions that included patient reminders and provider assessment and feedback. The cost per person screened was $134, however, in a clinic that included patient incentives and patient navigation in addition to patient reminders (30). In contrast, some studies have reported that patient navigation resulted in cost savings, especially for endoscopic facilities (31,32). A study that compared patients who were navigated to a screening colonoscopy with non-navigated patients at 1 endoscopy clinic found that navigated patients were significantly more likely to complete colonoscopy and to have adequate bowel preparation. The group of navigated patients also had significantly fewer no-shows and cancellations than the group of non-navigated patients (33). A business case has been made to support patient navigation in some clinical systems that led to increased revenues because of increased patient retention, physician loyalty, reduction in emergency department visits, hospitalizations, and reduced burdens on oncology providers (34). Some of these benefits of patient navigation, however, might not be immediate and might not be assessed. If they are assessed, benefits might not be attributed to patient navigation. As most CRCCP clinics are Federally Qualified Health Centers that might not realize many of the potential economic benefits because patients often go to endoscopy providers. CDC is planning to conduct comparative effectiveness studies to further elucidate cost-effectiveness and other barriers to implement patient navigation. For now, reimbursement through health insurers might be required to increase the scalability of this strategy in primary care settings serving populations most likely to benefit from patient navigation.

Many of the strategies that clinics are implementing, including provider reminders, patient reminders, provider assessment and feedback, and small media, have the potential to promote CRC screening for all patients, and they were associated with screening rate improvements in the first year of the CRCCP (18). With the CRCCP focus on Federally Qualified Health Centers that serve populations with high disease burden, strategies also have the potential to reduce cancer disparities. Patient navigation in particular can focus on patients who have substantial barriers to CRC screening and the least access to care (9,35). This intervention strategy, therefore, is uniquely suited to reduce cancer disparities. Cancer disparities reduction was demonstrated in a statewide CRC screening program in Delaware, population 982,895: 23% Black residents and 69% White residents. The Delaware program included financial coverage for CRC screening, treatment, and patient navigation by nurse coordinators. Statewide CRC screening rates increased from 48% among Black residents and from 58% among White residents in 2001 to 74% in both groups in 2009, and the program resulted in reduced disparities in CRC incidence and mortality (36). Future program evaluations could take a population health equity approach (36,37) by examining patient data of CRCCP clinics to determine if program strategies reduced disparities in CRC screening, stage at diagnosis, incidence, mortality, and which specific strategies contributed to the reduction in CRC disparities. Another set of analyses could examine trends in cancer disparities in the catchment areas of participating clinics during the implementation of CRCCP. An analysis that takes a population health equity approach would add a new perspective to the CRCCP program evaluation and provide crucial information on the value of all program strategies, including EBIs and patient navigation, in reducing CRC disparities. Further research is needed to gain a better understanding of the reasons clinics decide to implement some strategies over others and reasons other strategies are discontinued. Data from these analyses could guide future initiatives to increase CRC screening at a population level.

Our study included a data set with a limited number of variables and did not assess theoretical constructs, such as those of the Consolidated Framework for Implementation Research that might explain uptake and sustainability. Furthermore, many clinics could not provide data on the racial and ethnic characteristics of patients, and we were not able to examine if those characteristics were related to the uptake of intervention strategies, particularly patient navigation. Future studies should assess theoretical constructs that are relevant for implementation to illuminate the determinants of implementation and sustainability (38). We also did not have information about the quality of EBI and SA implementation, which likely varies considerably across clinics. Although respondents were encouraged to consult with their team, surveys were completed by one person per clinic who might not have had complete information. Responses may be influenced by respondent role in the clinic (eg, CRC champion versus a quality improvement specialist) and might also suffer from social desirability bias. Respondents were instructed to not report reducing structural barriers as a patient navigation activity, but it is possible that some respondents conflated these 2 strategies, because patient navigators often conduct work related to reducing structural barriers. Finally, CDC’s mandate that clinics implement at least 2 priority EBIs could have dictated to some extent the selection of strategies (priority EBIs versus optional SAs).

Our analysis focused on the uptake of 8 different strategies, all recommended by the Community Preventive Services Task Force, in a large number of clinics. Those clinics chose which EBIs and SAs to implement in the context of seeking to meet CRCCP program requirements. Primary care clinics participating in the CRCCP significantly increased implementation of 4 priority EBIs (patient reminder systems, provider reminder systems, provider assessment and feedback, and activities to reduce structural barriers) and 2 optional SAs (provider education and small media) to increase CRC screening over the first 3 program years. Uptake may be facilitated through technical and financial support provided by CRCCP awardees and integration of these strategies into clinic electronic health records systems. Implementation of patient navigation and community health workers remained flat over time, likely due, in part, to the need for ongoing funding for staff. Although use of patient navigation and community health workers may be effective strategies for reaching a clinic’s most underserved patients, additional support or encouragement may be required for clinics to add these services.
